# Three genes expressing Kunitz domains in the epididymis are related to genes of WFDC-type protease inhibitors and semen coagulum proteins in spite of lacking similarity between their protein products

**DOI:** 10.1186/1471-2091-12-55

**Published:** 2011-10-11

**Authors:** Adam Clauss, Margareta Persson, Hans Lilja, Åke Lundwall

**Affiliations:** 1 Lund University, Department of Laboratory Medicine, Clinical Chemistry, Skåne University Hospital, SE-205 02 Malmö, Sweden; 2 Departments of Clinical Laboratories, Surgery, and Medicine, Memorial Sloan-Kettering Cancer Center, 1275 York Avenue, New York, NY, 10065, NY, USA; 3 Karolinska Institute, Department of Oncology-Pathology, Karolinska Hospital, SE-171 76 Stockholm, Sweden

## Abstract

**Background:**

We have previously identified a locus on human chromosome 20q13.1, encompassing related genes of postulated WFDC-type protease inhibitors and semen coagulum proteins. Three of the genes with WFDC motif also coded for the Kunitz-type protease inhibitor motif. In this report, we have reinvestigated the locus for homologous genes encoding Kunitz motif only. The identified genes have been analyzed with respect to structure, expression and function.

**Results:**

We identified three novel genes; *SPINT3, SPINT4 *and *SPINT5*, and the structure of their transcripts were determined by sequencing of DNA generated by rapid amplification of cDNA ends. Each gene encodes a Kunitz domain preceded by a typical signal peptide sequence, which indicates that the proteins of 7.6, 8.7, and 9.7 kDa are secreted. Analysis of transcripts in 26 tissues showed that the genes predominantly are expressed in the epididymis. The recombinantly produced proteins could not inhibit the amidolytic activity of trypsin, chymotrypsin, plasmin, thrombin, coagulation factor Xa, elastase, urokinase and prostate specific antigen, whereas similarly made bovine pancreatic trypsin inhibitor (BPTI) had the same bioactivity as the protein isolated from bovine pancreas.

**Conclusions:**

The similar organization, chromosomal location and site of expression, suggests that the novel genes are homologous with the genes of WFDC-type protease inhibitors and semen coagulum proteins, despite the lack of similarity in primary structure of their protein products. Their restricted expression to the epididymis suggests that they could be important for male reproduction. The recombinantly produced proteins are presumably bioactive, as demonstrated with similarly made BPTI, but may have a narrower spectrum of inhibition, as indicated by the lacking activity against eight proteases with differing specificity. Another possibility is that they have lost the protease inhibiting properties, which is typical of Kunitz domains, in favor of hitherto unknown functions.

## Background

The mammalian semen coagulum proteins are a heterogeneous collection of proteins secreted at very high concentration by the seminal vesicles. There are two homologous semen coagulum proteins, denoted semenogelin I (SEMG1) and semenogelin II (SEMG2), in most primates [1-3]. Duplication of tandem repeats of 60 amino acid residues in both SEMG1 and SEMG2 is responsible for most of the size heterogeneity of semenogelin molecules in primates, but the frequent occurrence of premature stop codons is also a contributing factor [1,4,5]. It has also been reported that the structure of semenogelin molecules can be affected by gene conversion [6].

The murine seminal vesicles secrete six proteins, Svs1-Svs6, at high concentration [7-9]. It has been shown that the genes of Svs2-Svs6, and SEMG1 and SEMG2 are homologous, in spite of lacking similarity between their protein products [10,11]. This apparent paradox is explained by rapid evolution of single exon that codes for most of the gene product. The homologous genes are composed of three exons, the first of which encodes the signal peptide and the very first residues of the secreted protein, the second codes for the remainder of the protein and also carries a few 3' non-translated nucleotides, whereas the third exon only has 3' non-translated nucleotides and holds the poly-adenylation signal. Sequence comparison shows that the first and last exons are conserved in these genes, whereas the second exon is not [10]. Differing selection of splice acceptor sites and species unique repeat expansion have been proposed to explain the diversity of the second exon [10].

The proposed evolutionary mechanisms suggest that the seminal vesicle-transcribed genes might be related to genes with similar organization that code for secreted, but structurally very different proteins. This hypothesis led to the discovery that the genes of the elastase inhibitors elafin and secretory leukocyte protease inhibitor (SLPI), and the predominating seminal vesicle-secreted proteins are homologous, in spite of lacking similarity between their protein products [12]. Elafin and SLPI belong to the whey acidic protein four-disulfide core (WFDC) family of small protease inhibitors, which interacts with cognate proteases by what is known as the standard mechanism, by which the protease-binding loop of the inhibitors interacts with the catalytic site of proteases in a similar way as substrates [13].

With the advent of nucleotide sequences from the Human Genome Project, it became possible to assign the chromosomal location of genes in detail. Analysis of the human semenogelin locus on chromosome 20q12-13.1 showed that *PI3*, the gene of elafin, and *SLPI *are flanking *SEMG1 *and *SEMG2 *and an extended analysis of the chromosomal region identified 12 additional, homologous genes encompassing WFDC motif [14,15]. Most of the WFDC genes and *Svs2*-*Svs6 *are conserved at the homologous chromosomal region in the mouse, which suggests that the semen coagulum proteins probably originate from a WFDC gene [16]. Many of the WFDC genes are with certain specificity expressed in the epididymis and display accelerated evolution, which might indicate that they are of importance in male reproduction [16].

Three of the novel genes with WFDC motifs, *WFDC8*, *SPINLW1*, and *WFDC6 *also carry the motif of Kunitz-type protease inhibitors [14]. Similar to the WFDC domain, the conserved Kunitz domain is present in several proteins that inhibit proteases by the standard mechanism [13]. A well-known and much studied example is the bovine pancreatic trypsin inhibitor (BPTI), also known as aprotinin and until recently marketed as an anti-bleeding drug under the name of Trasylol^®^. Kunitz domains are also present in important protease inhibitors, such as the tissue factor pathway inhibitor, which regulates blood coagulation, and the light chain, also known as bikunin, of the multifunctional inter-alpha trypsin inhibitor [17-19].

We have in several previous studies, as outlined above, demonstrated that many of the genes at the semenogelin/WFDC locus are expressed in the male reproductive tract and display accelerated evolution by a variety of mechanisms. Most compelling are the findings suggesting that a WFDC gene gave rise to the genes of the semen coagulum proteins, as it also rises the question whether there could be still more genes at the locus on chromosome 20 that have gone through a similar metamorphosis. Our finding that the protease inhibitor locus on chromosome 20 carries several genes that code for inhibitors containing WFDC motifs only, while other genes encode products containing both WFDC and Kunitz motifs, raised the question as to whether there also exists homologous genes encoding putative inhibitors with Kunitz motifs alone. In this report, we have searched the locus for novel genes encoding proteins with Kunitz motifs. The identified genes have been analyzed with respect to structure, expression and function.

## Results

### Identification of three novel genes encoding Kunitz domains

**Figure 1:**
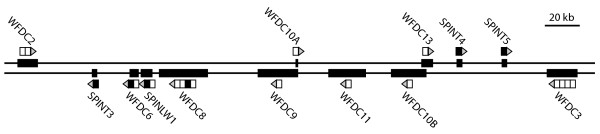
**Potential Kunitz inhibitors at the human WFDC locus**. The two DNA-strands of the telomeric sublocus of the WFDC/Semenogelin locus on human chromosome 20 are displayed as horizontal bars. The location of genes is indicated by thicker bars with arrow heads above or below, which illustrate WFDC domains in white, Kunitz domains in black and signal peptide in grey.

The nucleotide sequence of 1.8 Mb centered on the 0.7 Mb WFDC locus on chromosome 20q13.1 was translated in 6 reading frames and screened for Kunitz motifs. A total of six Kunitz motifs were detected, three of which were associated with proteins containing both Kunitz and WFDC motifs; the previously characterized SPINLW1, WFDC8 and WFDC6 [14,20]. The human WFDC locus consists of two subloci and the six genes encoding Kunitz motifs are all located at the telomeric sublocus (Figure 1). The first of the novel genes, *SPINT3*, is located 34 kb on the telomeric side of *WFDC2 *and 19 kb on the centromeric side of *WFDC6*. We located *SPINT4 *and *SPINT5 *next to each other, separated by 25 kb of intergenic DNA, with *SPINT4 *located 20 kb on the telomeric side of *WFDC13 *and *SPINT5 *25 kb on the centromeric side of *WFDC3*.

### Structure of transcripts

The nucleotide sequences of full-length transcripts of the novel genes were obtained from overlapping sequences generated by 3', and 5' RACE with cDNA from epididymis. The procedure yielded a single transcript for *SPINT3*, whereas two transcripts were associated with *SPINT4 *and presumably three with *SPINT5*. All genes give rise to transcripts that encode a Kunitz domain preceded by a signal peptide of 24 amino acid residues, indicating that the protein products are secreted. There are no signals for N-linked glycosylation in the primary structures, which suggests that the proteins are secreted without carbohydrate chains. Sizes of exons and introns are given in Table 1.

**Table 1:** Exon and intron sizes

Gene	Exons (bp)	Introns (bp)
Human SPINT3	92; 384	2,688

Human SPINT4A	134; 178; 68	1,397; 1,571

Human SPINT4B	134; 178; 201	1,397; 1,571

Mouse Spint5	170; 9; 170; 94	571; 940; 810

Human SPINT5	142; **53**; 169; 118	898; **547**; 1,120

Human SPINT5 alt 5'	**50**	**547**

Human SPINT5 alt 3'	66; 236; 202; 179; 85	1,473; 8,037; 130; 1,917; 2,830

Exons were identified by sequence comparison between transcripts and the reference genomic DNA in the Ensembl database (http://www.ensembl.org/index.html). RACE products generating alternative 5' and 3' ends are indicated with alt 5' and alt 3' respectively. Overlapping exon and intron sequences are written with bold fonts.

**Figure 2:**
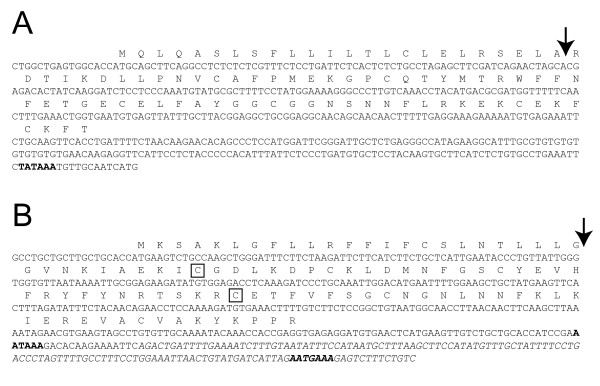
**Structure of SPINT3 and SPINT4**. The nucleotide sequences of transcripts are given with translation written above for (A) *SPINT3 *and (B) *SPINT4*. The arrows indicate location of the predicted signal peptide cleavage sites. The two Cys in *SPINT4*, which might form an unconventional disulphide bond, are boxed. The nucleotides present in the longer *SPINT4 *transcript, but not in the shorter, are italicized.

The *SPINT3 *transcript of 476 bp [GenBank: AY372172] terminates in a poly-A tail that is preceded by an unconventional poly-adenylation signal, TATAAA, which notably is identical to the canonical TATA-box sequence in eukaryotic genes (Figure 2A). It gives rise to a polypeptide of 89 amino acid residues that will be processed to a secreted protein of 7.6 kDa. The gene is not preceded by a conventional TATA-box, but there is an A and T rich sequence, TAAAAT, 26 bp upstream to the transcription initiation site.

The two *SPINT4 *transcripts are 380 [GenBank: AY372173] and 514 [GenBank: AY372174] nucleotides respectively. The difference between the transcripts is found in the 3' non-translated part, which in the smaller transcript consists of 61 nucleotides that overlap with those in the longer transcript of 194 nucleotides (Figure 2B). The conventional poly-adenylation signal, AATAAA, used in the shorter transcript, is skipped in the longer transcript for an unconventional poly-adenylation signal, AATGAAA, located further downstream. Both transcripts give rise to a polypeptide of 99 amino acid residues, which is processed to a protein of 8.7 kDa, encompassing a Kunitz motif that is lacking the fifth Cys of the consensus motif. Normally this would yield a free thiol group in the third Cys, the binding partner of the fifth Cys in the motif. However, there is another Cys located upstream to the Kunitz motif that potentially can form a disulphide with the third Cys of the motif (Figure 2B). There is a TATA-like sequence, TATAAC, preceding the gene by 25 bp.

**Figure 3:**
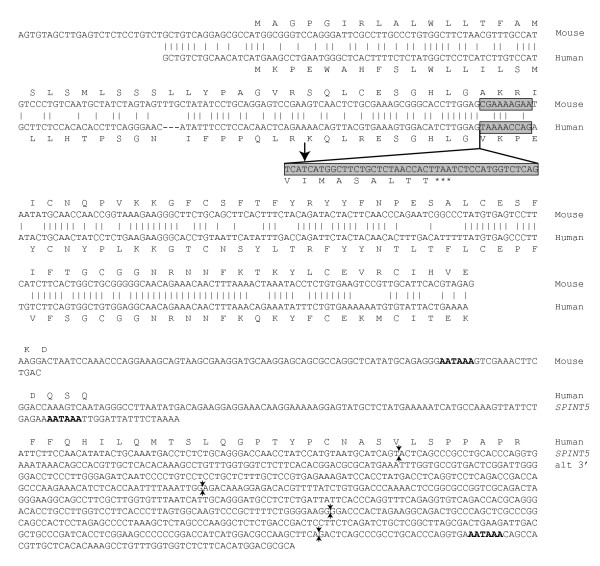
**Conservation of SPINT5**. The mouse and human SPINT5 transcripts were aligned with the computer program ALIGN. The nucleotide sequences that correspond to exon 1-3 of mouse and human *SPINT5 *are shown with identities indicated by vertical bars and gapped nucleotides by dashes. The second exon is shaded, with non-aligning nucleotides in the human gene inserted below; note the CAG at the end of the insertion that might indicate splice acceptor site, and the arrow that indicates starting nucleotide of the shorter 5' RACE product. The 3' ends of mouse *Spint5*, human *SPINT5*, and alternative 3' transcript of human *SPINT5 *are given at the bottom of the figure with poly-adenylation signals written in bold; location of introns in the alternative *SPINT5 *3' end are indicated with opposing arrows.

It was more difficult to obtain RACE products from *SPINT5 *than from the other two novel human Kunitz genes, but two transcripts were generated each by 3' RACE and 5' RACE when run with nested primers. The amplification problems suggests very low mRNA levels and the structures of the transcripts, which include premature stop codon and extensively many 3' non-translated exons, indicated that they might be spurious and not fully processed. Therefore, in another set of experiments, the Kunitz motif of *Spint5 *was identified *in silico *in the mouse genome. Following RT-PCR detection of the transcript in mouse testis, RACE, with testicular cDNA and primers based on the genomic sequence, yielded overlapping DNA fragments from which we could determine the complete structure of mouse *Spint5 *[GenBank: AY542490]. The mouse transcript of 443 bp is derived from four exons, two of which are located upstream to the exon coding for the Kunitz domain (Figure 3). In contrast to the human, the mouse transcript can be translated to yield a pre-protein of 105 amino acid residues that contains both a predicted signal peptide and the Kunitz domain. Removal of the signal peptide gives rise to a secreted protein of 9.1 kDa. The human and mouse transcripts differ with respect to the splicing of the second exon, which is only nine nucleotides in the mouse and codes for three amino acid residues. The splice acceptor site of the mouse transcript is structurally conserved in the human genome and, if used, it could generate a translation product encompassing both a signal peptide and a secreted protein of 9.7 kDa with a Kunitz domain (Figure 3).

The RACE procedure also yielded products for alternative 5' and 3' ends of human SPINT5. The sequence of a shorter 5' RACE product overlaps with the human transcript that is orthologous with mouse Spint5, but starts three nucleotides downstream of the splice acceptor site of the second exon (Figure 3). This transcript could potentially generate an intracellular Kunitz domain, as it is lacking the coding information of the signal peptide. The alternative 3' end is longer and derived from 5 exons located 3' to the exon with the Kunitz motif.

### Expression of transcripts in normal human tissues

Gene expression was analyzed by RT-PCR using RNA from a panel of 26 tissue specimens that previously had been screened for genes encoding WFDC domains [14]. Tissues that generated PCR products with the correct size of spliced transcripts were taken to quantitative analysis using real time PCR. The housekeeping genes *CSTB *and *APRT*, which were used for normalization of the PCR data, generated transcripts with melting temperatures of 80°C and 81.5°C and yielded Ct values in the range of 23-25 and 25-27 respectively. *SPINT3 *and *SPINT4 *gave rise to transcripts with melting temperatures of 76.5°C and 74°C and with cDNA from epididymis they generated Ct values that were similar or slightly lower than those of *CSTB*, but for the remainder of tissues the Ct values were 32 or higher; in a few tissues transcript was not even detectable. Different primer combinations were tested in order to measure the expression of *SPINT5*, but a detectable and specific signal was only obtained with a primer pair that primed in the Kunitz domain and in the last exon of the transcript; it yielded a Ct value of 35-36 in the epididymis. The efficacy of the PCR reactions was analyzed by linear regression of Ct values generated by serial dilution of epididymal cDNA (Table 2). An efficacy of 2 was assumed for *SPINT5*, as low levels prohibited analysis of its transcript by linear regression. Calculation of the relative expression shows that the level of *SPINT3 *and *SPINT4 *transcripts in the epididymis exceeds those in other tissues by more than three orders of magnitude (Table 3).

**Table 2:** PCR efficiency

Gene	Ct value range	Slope	R^2^	PCR efficiency
*APRT*	23-31	-3.14	0.996	2.08

*CSTB*	20-29	-3.35	0.996	1.99

*SPINT3*	20-29	-3.26	0.998	2.02

*SPINT4*	21-28	-3.48	0.997	1.94

The efficiency of PCR experiments was calculated by regression analysis of Ct values generated by serial dilution of epididymis cDNA.

**Table 3:** Tissue specific expression.

		Relative transcript levels (%)			
		
		Gene used for normalization			
**Tissue**	**Gene**	***APRT***	σ	***CSTB***	σ

Breast	*SPINT3*	0,010	0,001	0,018	0,002
	
	*SPINT4*	0,006	0,002	0,010	0,004

Epididymis	*SPINT3*	45	5	45	7
	
	*SPINT4*	100	14	100	18
	
	*SPINT5*	0,003	0,0005	0,003	0,0006

Kidney	*SPINT3*	0,0006	0,0002	0,002	0,0004
	
	*SPINT4*	0.006	0,002	0,016	0,002

Prostate	*SPINT3*	0,0007	0,0005	0,001	0,0007
	
	*SPINT4*	0,001	0,001	0,002	0,002

Seminal vesicle	*SPINT3*	0,056	0,017	0,053	0,014
	
	*SPINT4*	0,015	0,004	0,014	0,003

Skin	*SPINT3*	0,029	0,007	0,053	0,013
	
	*SPINT4*	0,003	0,001	0,005	0,002

Thyroid	*SPINT3*	0,004	0,003	0,005	0,004
	
	*SPINT4*	0,012	0,004	0,013	0,004

Testis	*SPINT3*	No signal	No signal	No signal	No signal
	
	*SPINT4*	0,004	0,004	0,008	0,007

Transcript levels of putative Kunitz inhibitors in normal tissues were analyzed by qRT-PCR. The transcript levels were calculated by the comparative ΔΔCt method and normalized with the housekeeping genes *APRT *and *CSTB*. Transcript levels are given as % of SPINT4 levels in the epididymis, which was set to 100%. *SPINT5 *was only detected in the epididymis.

### Recombinant expression and functional analysis

**Figure 4:**
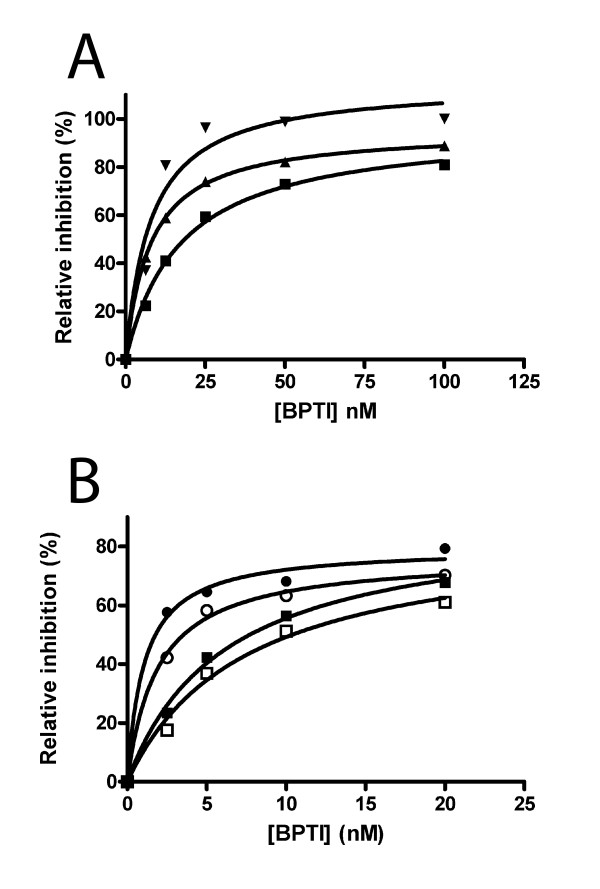
**Protease inhibition with recombinant BPTI**. (A) Serial dilutions of recombinant BPTI were incubated with 20 nM bovine chymotrypsin (■), 50 nM human plasmin (▼), and 5 nM bovine trypsin (▲). (B) Recombinant BPTI (filled symbols) and BPTI isolated from bovine pancreas (open symbols) were incubated with 10 nM (circles) and 20 nM (squares) bovine chymotrypsin. Residual amidolytic activity was measured and compared with samples without added inhibitor

Recombinant expression yielded proteins of around 27 kDa, which contain the Kunitz domains fused to vector encoded thioredoxin. Experiments with BPTI and BSTI, which served as positive controls, yielded around 5 mg of recombinant protein from 1 L of cultured bacteria after purification on Ni-chelate column. The isolated proteins were highly pure and appeared as single bands on SDS-PAGE under both reducing and non-reducing conditions. The isolated proteins were functionally active, as demonstrated by the inhibition of trypsin, plasmin and chymotrypsin by approximately equimolar concentrations of BSTI or BPTI (Figure 4A). A comparison with commercially available BPTI isolated from bovine pancreas showed that the recombinant protein inhibited chymotrypsin equally well (Figure 4B).

**Figure 5:**
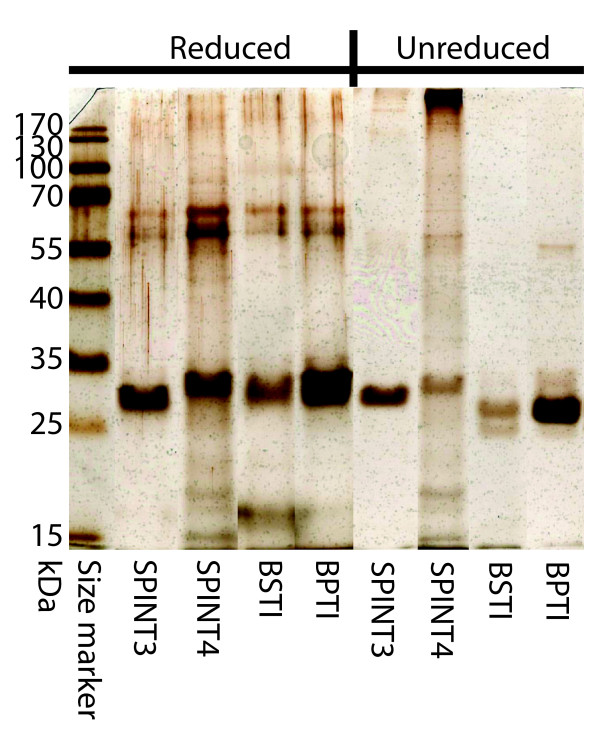
**SDS-PAGE**. Purified recombinant proteins were separated by SDS-PAGE and then silver stained. Masses of the molecular standard proteins in kDa are given to the left.

Experiments with SPINT3 and SPINT4 generated recombinant proteins in yields that were similar to those of BPTI and BSTI. Almost all recombinant SPINT3 was in a monomeric form, whereas this was the case for only a fraction of SPINT4 (Figure 5). Repeated experiments to produce recombinant SPINT5 in the same way failed and no recombinant protein could be isolated with the Ni-chelate column.

Recombinant SPINT3 and SPINT4 were tested on a panel of eight proteases with different specificity, in order to identify target enzymes. The recombinant proteins were tested at different molecular ratios of enzyme and inhibitor, but none of them displayed inhibitory activity towards the proteases in the panel at up to 1000-fold molar excess.

## Discussion

In this report, we describe the transcripts of three novel genes encoding Kunitz motif. They were discovered by analysis of nucleotide sequences from a locus where we previously have identified homologous genes of semen coagulum proteins and putative WFDC-type serine protease inhibitors [14]. In earlier studies we have shown that genes of different semen coagulum proteins and WFDC-type protease inhibitors are homologous, in spite of lacking structural similarity of their protein products [10-12,15]. By and large, these similarly organized genes carry the signal peptide, the secreted protein and 3' non-translated nucleotides on three separate exons. Sequence and size conservation of the first and the third exon suggests that the genes are homologous, whereas the second exons, encoding the secreted protein, in most instances display very little sequence conservation. Two mechanisms, de novo selection of splice sites and size expansion by duplication of repeats in combination with replication slippage, have been proposed to explain the structural variation of the second exon [10].

The novel genes of putative Kunitz inhibitors are organized in a similar way as the semen coagulum and WFDC genes. Another characteristic feature of the genes is that the splice acceptor of the second exon always is that of a phase 1 exon, which means that it starts with the second nucleotide of the codon. Of the novel genes, it is only *SPINT4 *that has all of the common features, whereas *SPINT3 *is lacking the third exon similar to *WFDC10A *and *WFDC10B*. At a first glance, human *SPINT5 *might appear to differ from the other genes at the locus, as the structure of the longer 3' RACE product suggests that it could be transcribed from 8 exons. However, the splice form that is orthologous with mouse *Spint5 *is very similar organized as *SPINT4*, except for the presence of the puzzling small second exon. In spite of the lacking relationship between the protein products, the similarities in gene organization, chromosomal location, and site of expression, suggests that the novel genes with Kunitz motif are homologous with genes encoding WFDC-inhibitors and semen coagulum proteins. A Kunitz inhibitor within the frame of a WFDC gene could have evolved by exon shuffling that created an intermediate encompassing both a WFDC and a Kunitz domain, as is present in SPINLW1 and WFDC6. A subsequent loss of the WFDC domain in a purifying process would have created a Kunitz gene with the same regulatory sequences as the original WFDC gene.

The problems we experienced in generating a full-length transcript of human *SPINT5 *were probably caused by low levels of transcript in the samples from our tissue panel. Furthermore, the in frame stop codon between the signal peptide and the Kunitz motif could indicate that the RACE products were emanating from either a not fully spliced nuclear transcript or a erroneously spliced cytoplasmic transcript. In order to address this, we generated the sequence of mouse *Spint5 *and compared it with those of the human transcripts. The failure to detect a mouse equivalent of the alternative human SPINT5 transcripts and the peculiar structure of them, with no coding information for a signal peptide or a 3' non-translated sequence derived from four exons, suggests that they could originate from not fully processed messages or cryptic transcripts generated by accidental transcription of the gene in a tissue where it is not normally expressed. The former could also be the case for the human transcript that is orthologous with the single mouse transcript, where the second exon might not have been fully processed, as it contains an in frame stop codon that prevents translation of the Kunitz motif. When the sequence of the second exon was shortened to the corresponding nine nucleotide of its mouse ortholog, the reading frame is kept and a Kunitz domain is translated from the third exon. The presence of an appropriately located CAG triplet that could function as splice acceptor in exon 2 raises the possibility that human *SPINT5 *could be processed the same way as mouse *Spint5*.

Semi-quantitative RT-PCR has previously shown that several of the WFDC genes at the locus on chromosome 20 are expressed in many different tissues [14]. However, the same study also showed that a number of the genes at the telomeric sublocus, *e.g*. *WFDC8, WFDC9, WFDC10A *and *WFDC13*, are transcribed in the epididymis with certain specificity. Epididymal specificity in expression has also been reported for *SPINLW1 *and *WFDC2 *[20,21]. In this study it is shown that *SPINT3 *and *SPINT4 *are highly specific for the epididymis as well. This conclusion is supported by qPCR that show expression levels in the epididymis that are of the same order of magnitude as for *CSTB*, a housekeeping gene with low to moderately high expression levels. More importantly, the transcript levels of *SPINT3 *and *SPINT4 *in the epididymis exceeds those of the same genes in other tissues that were analyzed by more than three orders of magnitude, which suggests that the expression in those tissues might be neglectable. The result is also in line with an earlier study aiming at identification of epididymis-specific genes in the mouse, in which *Spint4 *was found to be highly expressed in a narrow segment encompassing distal caput and proximal corpus of epididymis [22].

Transcripts of *SPINT5 *were only detected in the epididymis, but at a level that is at least four orders of magnitude lower than for *SPINT3 *and *SPINT4*. In analogy with the discussion above, the low transcript level would indicate that under normal conditions *SPINT5 *presumably is of less importance in the tissues that were analyzed in this study. From this follows that *SPINT5 *probably is expressed either at a relatively rare anatomical site or at a developmental stage that is not covered by this investigation. In ruminants, it has been shown that several proteins with Kunitz motifs are expressed and secreted by the conceptus prior to, or at the time of, trophoblast implantation [23,24]. Although, there are major differences in the implantation process between ruminants and many other mammals, e.g. in the timing, it is not unlikely that Kunitz inhibitors could be expressed at the time of trophoblast implantation also in humans, and one interesting candidate to study would be *SPINT5*.

Protein domains are building blocks that usually fold to explicit three dimensional structures, which many times have a specific function. The Kunitz domain consists of 50-60 amino acid residues, six of which are Cys that form three disulphide bonds and have characteristic locations in the primary structure. Studies on recombinant Kunitz domains expressed in *E. coli *show that proper folding and disulphide formation can be achieved either by directing the recombinant protein to the periplasmic space or by refolding of the protein extracted from inclusion bodies [17,25]. Our approach was to express the recombinant protein as a part of a larger fusion product with thioredoxin, that would generate high solubility and support the proper folding of the Kunitz domain, and by using the *E. coli *K-12 sub-strain Origami(DE3), formation of disulphide bonds in the cytoplasm would be favored. Studies with recombinant BPTI and BSTI showed that the proteins isolated from the cytoplasm were almost exclusively in a monomeric state, which suggests correct disulphide formation and folding of the Kunitz domain. Furthermore, the recombinant inhibitors were as functionally active as BPTI isolated from bovine pancreas, something that also indicates that they are correctly folded. Expression of SPINT3 yielded an exclusively monomeric protein in a similar way as BPTI and BSTI, which suggests that the Kunitz domain was properly folded. Recombinant expression of SPINT4 yields high molecular mass complexes that probably consist of misfolded peptide chains held together by disulphide bonds in an unorganized way, but also a substantial fraction of monomers that presumably are correctly folded. The structure of SPINT4 differs from that of the consensus Kunitz domain. The latter contains three conserved disulphide bridges, but in SPINT4 there will only be two, as one Cys is missing. The absent disulphide would have linked the C-terminal alpha-helix to one of the beta-strands at the core of the molecule. Earlier experiment has shown that the folding of the Kunitz domain is only minimally affected by removal of this bridge and would not affect the protease-binding loop that is situated at the opposite side of the molecule [26]. The location of a Cys amino-terminal to the Kunitz domain in SPINT4 suggests that the monomeric form will have either two free thiol groups, or more likely a disulphide bridge between them. The crystal structure of BPTI shows that the amino-terminus of the protein is located relatively close to the third Cys of the Kunitz motif. Therefore, it is not unlikely that a disulfide bridge could be formed in SPINT4 without affecting the protease-binding loop, which is located at the other end of the molecule. This would create a strand of 6 amino acid residues that links the protein's amino-terminal part to the third Cys of the Kunitz motif.

Despite several attempts, we did not succeed to isolate recombinant SPINT5 from the cytoplasm of Origami(DE3) cells. It is not clear whether the failure was due to inherent instability of the protein product or whether the problems are due to technical problems with our construct, but future studies will presumably give an answer.

**Figure 6:**
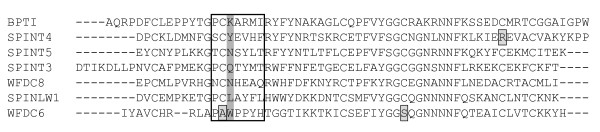
**Conservation of the Kunitz domain**. Exons encoding Kunitz domains were translated and the resulting peptide sequences were aligned using CLUSTALW. The amino acid residues homologous with those of the peptidase binding loop (P3-P'3) in BPTI are framed and the P1 position is shaded. Positions with mutated Cys in SPINT4 and WFDC6 are framed and shaded.

The experiments with recombinant BPTI demonstrated, not only that the Kunitz domain can be correctly folded in Origami(DE3) cells, but also that the fusion product with thioredoxin could be an equally efficient protease inhibitor as BPTI isolated from a natural source. It is therefore likely that recombinant SPINT3 and SPINT4 would be functional as well. They were tested by a set of proteolytic enzymes that represent different catalytic specificities, but unfortunately, we did not succeed to identify a target protease. The amino acid residues in BPTI that interact with target proteases have been characterized in detail and homologous residues can be identified in other Kunitz domains by sequence alignment [27]. There are several residues that are important for protease-binding, but those flanking the scissile bond, particularly the residue at the P1 position, are vital for the target- protease specificity [28,29]. The probable scissile bonds in SPINT3 and SPINT4 are between Gln and Thr, and Tyr and Glu, respectively (Figure 6). One might then have expected both of them to inhibit PSA, which has been demonstrated to cleave C-terminal to both Gln and Tyr in SEMG1 and SEMG2 [30]. There was no inhibition and perhaps the interaction with the inhibitor protein was prevented by the kallikrein loop in PSA, which restricts the access to the catalytic cleft [31]. In chymotrypsin there is no such structure as the kallikrein loop and SPINT4 could be predicted to interact with this enzyme, which cleaves after large hydrophobic residues. Under our experimental condition there was no inhibition, which suggests that there is no high affinity interaction between chymotrypsin and SPINT4. This emphasizes the importance of contact between amino acid residues other than those of the scissile bond, which is nicely shown with the inhibition of chymotrypsin by BPTI; the scissile bond in this case is between Lys and Ala, i.e. residues that would predict specificity for trypsin, but not chymotrypsin. In spite of this, chymotrypsin is inhibited by BPTI.

The WFDC genes at the locus on chromosome 20 could be important during sperm maturation, as several of them are highly expressed in the epididymis. There is this far no report on target proteases in the epididymis. It has also been speculated that the WFDC genes are important but not fully recognized components, of innate immunity; in which some modulate host response by regulating endogenous pro-inflammatory proteases, such as elastase and cathepsin G, whereas others regulate poorly characterized exogenous proteases used for tissue invasion by microbes [16]. The genes expressing Kunitz domains could have a similar function. In this report it is shown that *SPINT3 *and *SPINT4 *with high specificity are expressed in the epididymis, which suggests a role in the maturation of spermatozoa. An innate immune function can also inferred, as it has been shown that the Kunitz domain of SPINLW1 inhibits elastase and display antibacterial activity [32].

## Conclusions

We have in this report described three genes that produce small secreted proteins, composed of single Kunitz domains that are relatively specific for the epididymis. The similar organization, chromosomal location and site of expression, suggests that the novel genes encoding Kunitz motifs are homologous with the genes of WFDC-type protease inhibitors and semen coagulum proteins, in spite of lacking similarity in primary structure of their protein products. A likely mechanism, by which the genes could have been formed, include an exon shuffling event, in which the Kunitz motif was taken up by a WFDC gene, and a following purifying process, in which the WFDC domain was lost. This would then be the third mechanism, beside de novo selection of splice site and repeat expansion, by which the regulatory frame of a conserved WFDC/semen coagulum gene could generate a new protein product. The restricted expression of the novel genes to the epididymis suggests that the secreted Kunitz domains are important in male reproduction. The recombinantly produced proteins are presumably bioactive, as demonstrated with similarly made BPTI, but may have a narrower spectrum of inhibition, as indicated by the lacking activity against eight proteases with differing specificity. Another possibility is that they have lost the protease inhibiting property, which is typical of Kunitz domains, in favor of new hitherto unidentified functions.

## Methods

### Identification of novel genes in genomic DNA sequences

The nucleotide sequence of the WFDC-locus on chromosome 20 was translated in six reading frames and scanned for short sequence motifs as previously described [14]. Kunitz domains were localized using the motif GCXGN and NNF (single letter amino acid code where × means any residue) and confirmed by identification of appropriately spaced Cys residues through manual inspection of the sequence.

### RT-PCR

Preparation of RNA samples and conditions for cDNA synthesis has been described earlier [15]. Primers were designed from nucleotide sequences originating from different exons to enable differentiation between PCR products from spliced transcripts and non-spliced transcripts or genomic DNA (Table 4). A panel of cDNA derived from different tissues were screened by PCR using previously described conditions [14]. The PCR products were analyzed by electrophoresis in 2% agarose gels stained by ethidium bromide. Samples that by this procedure generated a PCR product from a spliced transcript were taken to quantitative PCR using real time detection.

**Table 4:** Oligonucleotide primers.

Target gene	Purpose	Primer sequence (5' to 3')
Human SPINT3	RT-PCR and 3' RACE	CCTCTCTCTCGTTTCTCCTGATTCTCACT

Human SPINT3	RT-PCR and 5' RACE	CGCAGCCTCCGTAAGCAAATAACTCACAT

Human SPINT3	qRT-PCR	TCAGGCCTCTCTCTCGTTTCTCCTGATT

Human SPINT3	qRT-PCR	CCCTTTTCCATAGGAAAAGCGCATACAT

Human SPINT4	RT-PCR and 3' RACE	CATCTTCTGCTCATTGAATACCCTGTTATT

Human SPINT4	RT-PCR and 5' RACE	TTACAGCCGGAGAAGACAAAAGTTTCACAT

Human SPINT4	qRT-PCR	TCTTCTGCTCATTGAATACCCTGTTATT

Human SPINT4	qRT-PCR	GAACTTCATAGCAGCTTCCAAAATTCAT

Human SPINT5	3' RACE	CAACTATCCTCTGAAGAAGGGCACCTGT

Human SPINT5	Nested 3' RACE	ATGTGAGCCCTTTGTCTTCAGTGGCTGT

Human SPINT5	5' RACE	GTTTCTGTTGCCTCCACAGCCACTG

Human SPINT5	Nested 5' RACE	CAGCCACTGAAGACAAAGGGCTCAC

Human SPINT5	qRT-PCR	GCTGTGGAGGCAACAGAAACAACTTTAA

Human SPINT5	qRT-PCR	CCTCCTTCTGTCATATTAAGGCCCTATT

Mouse Spint5	RT-PCR and 3' RACE	CCTGCAGGAGTCCGAAGTCAACTCTGCGA

Mouse Spint5	RT-PCR and 5' RACE	TGCAACGGACTTCACAGAGGTATTTAGT

Human APRT	qRT-PCR	CTTGGACTGGGCTGCGTGCTCATCCGAA

Human APRT	qRT-PCR	GTCCTGGCTCCAGGGCGTCTTTCTGAAT

Human CSTB	qRT-PCR	AGTTCCCTGTGTTTAAGGCCGTGTCATT

Human CSTB	qRT-PCR	GTTTTCATGAGGGAGAGATTGGAACACT

Human SPINT3	Cloning in pET32b	GGATCCGAATTCGCGAGACACTATCAAGGATCTCCTCCCAAAT

Human SPINT3	Cloning in pET32b	TCGAGTGCGGCCGCTCAGGTGAACTTGCAGAATTTCTCACAT

Human SPINT4	Cloning in pET32b	CGAATTCGGGTGGTGTTAATAAAATTGCGGAGA

Human SPINT4	Cloning in pET32b	GCGCGGCCGCCTATTGACTTTGGTCCTTTTCAG

Human SPINT5	Cloning in pET32b	CAGTGAATTCCGAATACTGCAACTATCCTCTGAAGAA

Human SPINT5	Cloning in pET32b	TGCAGCGGCCGCTACTTTTCAGTAATACACATTTTTTCACAGA

Bovine BPTI	Cloning in pET32b	GGATCCGAATTCGCCTGACTTCTGCCTAGAGCCTCCATAT

Bovine BPTI	Cloning in pET32b	TCGAGTGCGGCCGCTCACCAGGGCCCAATAGCACCACCACAGGT

The underlined sequences are not present in the target genes.

### Quantitative RT-PCR (qRT-PCR)

The cDNA for qRT-PCR was made from DNAase-free RNA using reagents and protocol provided by the supplier (Fermentas, Helsingborg, Sweden). Briefly, 3 μg of RNA was treated at 37°C for 30 min with 1 unit of RNase-free DNase in 10 μl of 10 mM Tris-HCl pH 7.5, 2.5 mM MgCl_2 _and 0.1 mM CaCl_2_. Following addition of 1 μl of 25 mM EDTA, the enzyme was inactivated by incubation at 65°C for 10 min. The material was then taken to oligo(dT)_18_-primed cDNA synthesis in a volume of 20 μl using the RevertAid H Minus First Strand cDNA Synthesis kit (Fermentas) for 1 h at 42°C. Finally, the reverse transcriptase was inactivated by heating to 70°C for 5 min, and the samples were diluted with H_2_O to 200 μl and stored at -20°C until used. Negative controls were produced in the same way, but with the reverse transcriptase omitted.

The PCR was done in MicroAmp Optical 384-Well Reaction Plates (Applied Biosystems, Stockholm, Sweden) using the 7900 HT Fast Real-Time PCR System (Applied Biosystems). PCR primers were selected such that the resultant transcript should be less than 150 bp (Table 4). The reaction mix was made from 3 μl of diluted cDNA, 2 μl of primers at 5 μM each, and 5 μl of Fast SYBR Green Master Mix (Applied Biosystems). Samples were analyzed in quadruplicates. The cycling conditions consisted of an initial activation step at 95°C for 20 s, followed by 40 cycles of denaturation at 95°C for 1 s and annealing and extension at 60°C for 20 s. Following PCR the homogeneity of the products were checked by DNA melting analysis. The real time PCR data were analyzed by the Sequence Detection System 2.3 software. The relative amount of transcripts was calculated by the comparative ΔΔCt method and normalized with values of the housekeeping genes *CSTB *and *APRT *[33,34]. The PCR efficiency was calculated from Ct values generated by serially diluted epididymis cDNA.

### Rapid amplification of cDNA ends (RACE)

Synthesis of RACE ready cDNA and touch down PCR was done with the SMART or the SMARTer RACE cDNA Amplification Kits (Clontech, In vitro, Stockholm, Sweden) and RNA from human epididymis or mouse testis, essentially as described [14]. The primers were the same as used for RT-PCR, except for experiment on human *SPINT5*, in which RACE products were generated with the SMARTer RACE cDNA amplification kit and two rounds of PCR using nested primers, as recommended in the protocol supplied with the kit. The RACE products were purified using JetQuick (Genomed, Saween Werner AB, Malmö, Sweden) or Nucleotrap (Clontech) and subjected to DNA sequencing using the Big Dye Terminator Cycle Sequencing Ready Reaction Kit 3.0 and a 3730 DNA Analyzer (Applied Biosystems). The sequences were analyzed with the Sequence Analysis 5.1 program (Applied Biosystems) and bioinformatics tools available at the EBI web server (http://www.ebi.ac.uk/Tools/). Translations of transcripts were screened for signal peptides using the SignalP 3.0 server [35]. Sequences were deposited with the Genbank Sequence Database and are available under accession numbers AY372172, AY372173, AY372174, and AY542490.

### Construction of expression plasmids

Transcripts for expression of Kunitz domains in the prokaryotic expression vector pET32b were generated by PCR using primers that contain restriction enzyme recognition sites for *EcoRI *and *NotI *(Table 4). The PCR protocol was the same as for RT-PCR and as template served human epididymal cDNA, and human and bovine genomic DNA. The PCR that was run in order to generate the control plasmid encoding BPTI also yielded transcript for the homologous bovine spleen trypsin inhibitor (BSTI) [36]. The restricted PCR products were ligated into plasmids using the Rapid DNA Ligation Kit (Fermentas) and different chemo-competent bacteria were transformed and grown with appropriate antibiotics. Plasmids were purified by QIAprep Spin MiniPrep Kit (Qiagen AB, Sollentuna, Sweden) from 1.5 ml of overnight cultures grown in LB medium containing appropriate antibiotics, and then digested with restriction enzyme and analyzed by electrophoresis in 2% agarose gels containing ethidium bromide (1 μg/ml). Plasmids containing inserts of correct size were verified by DNA sequencing.

### Protein expression

Origami(DE3) bacteria containing plasmids designed for prokaryotic expression were grown at 37°C in 500 ml of LB medium containing antibiotics until mid-logarithmic phase. The culture was chilled and the synthesis of recombinant protein was induced by addition of isopropyl-β-D-thiogalactopyranoside (IPTG) to a final concentration of 1 mM, where after growth continued at 30°C for 5 hours. Bacteria were harvested by centrifugation at 10,000 × *g *for 10 minutes at 4°C. The resulting pellet was washed in 50 ml of 20 mM Tris-HCl, 0.5 M NaCl, pH 7.5 and then resuspended in the same buffer containing 1 mg/ml of lysozyme. The suspension was made 1 mM with respect to PMSF and then incubated on ice for 30 min. Next, the bacterial suspension was sonicated with an Ultrasonic processor XL 2020 (Misonix inc., Farmingdale, NY, USA) for 30 minutes with 10 s burst separated by intervals of 10 s, using the power setting 3. Cell debris was removed by centrifugation at 16,000 × *g *for 20 min. The supernatant was dialyzed against 50 mM Na-phosphate pH 8.0, 0.3 M NaCl and then passed through a 0.45 micron filter (Millipore AB, Solna, Sweden).

The recombinant proteins were purified on a 12 ml column of Ni-NTA superflow (Qiagen) that was run at 2 ml/min in 50 mM Na-phosphate, 0.3 M NaCl and 10 mM imidazole, pH 8.0. Proteins were eluted by increasing the concentration of imidazole to 100 mM or 300 mM.

### Protease inhibition

The amidolytic activity of proteases was measured with purified enzymes and synthetic peptide substrate. The assays were run in microtiter plates with enzyme, inhibitor and peptide substrate in wells containing 100 μl of 50 mM Tris-HCl, pH 8.0, 0.15 M NaCl and 0.05% Tween 20. Enzymatic activity was measured at 37°C in a Viktor^2 ^instrument (Wallac, Upplands Väsby, Sweden) and displayed as the time-dependent increase in absorbance at 405 nm. Inhibitors at various concentrations were incubated with proteases for 15 min at 37°C in the assay buffer. Reactions were initiated by addition of peptide substrate. The following concentrations of proteases (purchased from Sigma-Aldrich Sweden AB, Stockholm, Sweden) were used: 5 nM of bovine trypsin, 10 and 20 nM of bovine chymotrypsin, 50 nM human plasmin, 10 nM human urokinase, 15 nM bovine thrombin, 15 nM bovine Factor Xa, 10 nm human leukocyte elastase, and 20 nM porcine pancreatic elastase. Analysis was also made with 10 nM human prostate-specific antigen (PSA), purified from human seminal plasma by affinity chromatography using Mab 2E9 [37]. The activity of trypsin, plasmin, urokinase, thrombin and Factor Xa were measured with the peptide substrates S-2366, pyroGlu-Pro-Arg-pNA (Chromogenix, Mölndal, Sweden), chymotryptic activity with S-2586, MeO-Suc-Arg-Pro-Tyr-pNA (Chromogenix,), elastase activity with N-Suc-Ala-Ala-Ala-pNA (VWR International AB, Stockholm, Sweden) and, PSA activity with Mu-Ser-Ser-Tyr-Tyr-AMC (custom made by MedProbe, Lund, Sweden). The inhibition was calculated as the decrease in enzymatic activity between samples pre-incubated with inhibitor and buffer only. The kinetic data was analyzed and displayed using the computer program GraphPad Prism, version 4.00 for Windows (GraphPad Software, San Diego CA, USA)

## Authors' contributions

AC designed and carried out experimental procedures and drafted the manuscript; MP designed and carried out experimental procedures, HL was involved in planning and design of experiments and reviewed and contributed substantive edits of the manuscript, ÅL initiated the study, identified the novel gens, contributed to the experimental design, made the qPCR and wrote the final manuscript. All authors have read and approved the final manuscript.

## References

[B1] Hurle B Swanson W Green ED Comparative sequence analyses reveal rapid and divergent evolutionary changes of the WFDC locus in the primate lineage Genome Res 2007 17 3 276 286 10.1101/gr.6004607 17267810 PMC1800918

[B2] Lilja H Abrahamsson PA Lundwall A Semenogelin, the predominant protein in human semen. Primary structure and identification of closely related proteins in the male accessory sex glands and on the spermatozoa J Biol Chem 1989 264 3 1894 1900 2912989

[B3] Lilja H Lundwall A Molecular cloning of epididymal and seminal vesicular transcripts encoding a semenogelin-related protein Proc Natl Acad Sci USA 1992 89 10 4559 4563 10.1073/pnas.89.10.4559 1584792 PMC49122

[B4] Jensen-Seaman MI Li WH Evolution of the hominoid semenogelin genes, the major proteins of ejaculated semen J Mol Evol 2003 57 3 261 270 10.1007/s00239-003-2474-x 14629036

[B5] Ulvsback M Lundwall A Cloning of the semenogelin II gene of the rhesus monkey. Duplications of 360 bp extend the coding region in man, rhesus monkey and baboon Eur J Biochem 1997 245 1 25 31 10.1111/j.1432-1033.1997.00025.x 9128720

[B6] Valtonen-Andre C Olsson AY Kullberg M Nayudu PL Lundwall A The Common Marmoset (Callithrix jacchus) Has Two Very Similar Semenogelin Genes as the Result of Gene Conversion Biol Reprod 2007 76 4 604 610 10.1095/biolreprod.106.057661 17192513

[B7] Higgins SJ Burchell JM Mainwaring WI Androgen-dependent synthesis of basic secretory proteins by the rat seminal vesicle Biochem J 1976 158 2 271 282 985427 10.1042/bj1580271 PMC1163968

[B8] Lundwall A Peter A Lovgren J Lilja H Malm J Chemical characterization of the predominant proteins secreted by mouse seminal vesicles Eur J Biochem 1997 249 1 39 44 10.1111/j.1432-1033.1997.t01-2-00039.x 9363751

[B9] Ostrowski MC Kistler MK Kistler WS Purification and cell-free synthesis of a major protein from rat seminal vesicle secretion. A potential marker for androgen action J Biol Chem 1979 254 2 383 390 762067

[B10] Lundwall A Lazure C A novel gene family encoding proteins with highly differing structure because of a rapidly evolving exon FEBS Lett 1995 374 1 53 56 10.1016/0014-5793(95)01076-Q 7589511

[B11] Lundwall A The cloning of a rapidly evolving seminal-vesicle-transcribed gene encoding the major clot-forming protein of mouse semen Eur J Biochem 1996 235 1-2 424 430 10.1111/j.1432-1033.1996.00424.x 8631362

[B12] Lundwall A Ulvsback M The gene of the protease inhibitor SKALP/elafin is a member of the REST gene family Biochem Biophys Res Commun 1996 221 2 323 327 10.1006/bbrc.1996.0594 8619854

[B13] Krowarsch D Cierpicki T Jelen F Otlewski J Canonical protein inhibitors of serine proteases Cell Mol Life Sci 2003 60 11 2427 2444 10.1007/s00018-003-3120-x 14625687 PMC11138524

[B14] Clauss A Lilja H Lundwall A A locus on human chromosome 20 contains several genes expressing protease inhibitor domains with homology to whey acidic protein Biochem J 2002 368 Pt 1 233 242 12206714 10.1042/BJ20020869 PMC1222987

[B15] Lundwall A Clauss A Identification of a novel protease inhibitor gene that is highly expressed in the prostate Biochem Biophys Res Commun 2002 290 1 452 456 10.1006/bbrc.2001.6224 11779191

[B16] Clauss A Lilja H Lundwall A The evolution of a genetic locus encoding small serine proteinase inhibitors Biochem Biophys Res Commun 2005 333 2 383 389 10.1016/j.bbrc.2005.05.125 15950183 PMC1939935

[B17] Burgering MJ Orbons LP van der Doelen A Mulders J Theunissen HJ Grootenhuis PD Bode W Huber R Stubbs MT The second Kunitz domain of human tissue factor pathway inhibitor: cloning, structure determination and interaction with factor Xa J Mol Biol 1997 269 3 395 407 10.1006/jmbi.1997.1029 9199408

[B18] Gebhard W Hochstrasser K Fritz H Enghild JJ Pizzo SV Salvesen G Structure of inter-alpha-inhibitor (inter-alpha-trypsin inhibitor) and pre-alpha-inhibitor: current state and proposition of a new terminology Biol Chem Hoppe Seyler 1990 371 Suppl 13 22 1698066

[B19] Petersen LC Bjorn SE Olsen OH Nordfang O Norris F Norris K Inhibitory properties of separate recombinant Kunitz-type-protease-inhibitor domains from tissue-factor-pathway inhibitor Eur J Biochem 1996 235 1-2 310 316 10.1111/j.1432-1033.1996.0310f.x 8631347

[B20] Richardson RT Sivashanmugam P Hall SH Hamil KG Moore PA Ruben SM French FS O'Rand M Cloning and sequencing of human Eppin: a novel family of protease inhibitors expressed in the epididymis and testis Gene 2001 270 1-2 93 102 10.1016/S0378-1119(01)00462-0 11404006

[B21] Kirchhoff C Habben I Ivell R Krull N A major human epididymis-specific cDNA encodes a protein with sequence homology to extracellular proteinase inhibitors Biol Reprod 1991 45 2 350 357 10.1095/biolreprod45.2.350 1686187

[B22] Penttinen J Pujianto DA Sipila P Huhtaniemi I Poutanen M Discovery in silico and characterization in vitro of novel genes exclusively expressed in the mouse epididymis Mol Endocrinol 2003 17 11 2138 2151 10.1210/me.2003-0008 12920233

[B23] Kramer KK Duffy JY Klemann SW Bixby JA Low BG Pope WF Roberts RM Selective cloning of cDNA for secretory proteins of early embryos. Identification of a transiently expressed kunitz domain protein from preimplantation sheep trophoblast J Biol Chem 1994 269 10 7255 7261 7510284

[B24] MacLean JA Chakrabarty A Xie S Bixby JA Roberts RM Green JA Family of Kunitz proteins from trophoblast: expression of the trophoblast Kunitz domain proteins (TKDP) in cattle and sheep Mol Reprod Dev 2003 65 1 30 40 10.1002/mrd.10262 12658631

[B25] Krokoszynska I Dadlez M Otlewski J Structure of single-disulfide variants of bovine pancreatic trypsin inhibitor (BPTI) as probed by their binding to bovine beta-trypsin J Mol Biol 1998 275 3 503 513 10.1006/jmbi.1997.1460 9466927

[B26] Eigenbrot C Randal M Kossiakoff AA Structural effects induced by removal of a disulfide-bridge: the X-ray structure of the C30A/C51A mutant of basic pancreatic trypsin inhibitor at 1.6 A Protein Eng 1990 3 7 591 598 10.1093/protein/3.7.591 1699222

[B27] Huber R Kukla D Bode W Schwager P Bartels K Deisenhofer J Steigemann W Structure of the complex formed by bovine trypsin and bovine pancreatic trypsin inhibitor. II. Crystallographic refinement at 1.9 A resolution J Mol Biol 1974 89 1 73 101 10.1016/0022-2836(74)90163-6 4475115

[B28] Castro MJ Anderson S Alanine point-mutations in the reactive region of bovine pancreatic trypsin inhibitor: effects on the kinetics and thermodynamics of binding to beta-trypsin and alpha-chymotrypsin Biochemistry 1996 35 35 11435 11446 10.1021/bi960515w 8784199

[B29] Otlewski J Jaskolski M Buczek O Cierpicki T Czapinska H Krowarsch D Smalas AO Stachowiak D Szpineta A Dadlez M Structure-function relationship of serine protease-protein inhibitor interaction Acta Biochim Pol 2001 48 2 419 428 11732612

[B30] Malm J Hellman J Hogg P Lilja H Enzymatic action of prostate-specific antigen (PSA or hK3): substrate specificity and regulation by Zn(2+), a tight-binding inhibitor Prostate 2000 45 2 132 139 10.1002/1097-0045(20001001)45:2<132::AID-PROS7>3.0.CO;2-3 11027412

[B31] Menez R Michel S Muller BH Bossus M Ducancel F Jolivet-Reynaud C Stura EA Crystal structure of a ternary complex between human prostate-specific antigen, its substrate acyl intermediate and an activating antibody J Mol Biol 2008 376 4 1021 1033 10.1016/j.jmb.2007.11.052 18187150

[B32] McCrudden MT Dafforn TR Houston DF Turkington PT Timson DJ Functional domains of the human epididymal protease inhibitor, eppin FEBS J 2008 275 8 1742 1750 10.1111/j.1742-4658.2008.06333.x 18331357

[B33] Eisenberg E Levanon EY Human housekeeping genes are compact Trends Genet 2003 19 7 362 365 10.1016/S0168-9525(03)00140-9 12850439

[B34] Bookout AL Cummins CL Mangelsdorf DJ Pesola JM Kramer MF High-throughput real-time quantitative reverse transcription PCR Curr Protoc Mol Biol 2006 Chapter 15 Unit 15 18 10.1002/0471142727.mb1508s73 18265376

[B35] Bendtsen JD Nielsen H von Heijne G Brunak S Improved prediction of signal peptides: SignalP 3.0 J Mol Biol 2004 340 4 783 795 10.1016/j.jmb.2004.05.028 15223320

[B36] Creighton TE Charles IG Sequences of the genes and polypeptide precursors for two bovine protease inhibitors J Mol Biol 1987 194 1 11 22 10.1016/0022-2836(87)90711-X 2441071

[B37] Piironen T Villoutreix BO Becker C Hollingsworth K Vihinen M Bridon D Qiu X Rapp J Dowell B Lovgren T Determination and analysis of antigenic epitopes of prostate specific antigen (PSA) and human glandular kallikrein 2 (hK2) using synthetic peptides and computer modeling Protein Sci 1998 7 2 259 269 9521101 10.1002/pro.5560070205 PMC2143911

